# Age‐dependent integrity of the meiotic spindle assembly checkpoint in females requires Aurora kinase B

**DOI:** 10.1111/acel.13489

**Published:** 2021-10-26

**Authors:** Cecilia S. Blengini, Alexandra L. Nguyen, Mansour Aboelenain, Karen Schindler

**Affiliations:** ^1^ Department of Genetics; Rutgers The State University of New Jersey Piscataway NJ USA; ^2^ Human Genetics Institute of New Jersey Piscataway NJ USA; ^3^ Department of Theriogenology Faculty of Veterinary Medicine Mansoura University Mansoura Egypt

**Keywords:** Aurora kinase B, infertility, reactive oxygen species, reproductive aging, spindle assembly checkpoint

## Abstract

A hallmark of advanced maternal age is a significant increase in meiotic chromosome segregation errors, resulting in early miscarriages and congenital disorders. These errors most frequently occur during meiosis I (MI). The spindle assembly checkpoint (SAC) prevents chromosome segregation errors by arresting the cell cycle until proper chromosome alignment is achieved. Unlike in mitosis, the SAC in oocytes is desensitized, allowing chromosome segregation in the presence of improperly aligned chromosomes. Whether SAC integrity further deteriorates with advancing maternal age, and if this decline contributes to increased segregation errors remains a fundamental question. In somatic cells, activation of the SAC depends upon Aurora kinase B (AURKB), which functions to monitor kinetochore–microtubule attachments and recruit SAC regulator proteins. In mice, oocyte‐specific deletion of AURKB (*Aurkb* cKO) results in an increased production of aneuploid metaphase II‐arrested eggs and premature age‐related infertility. Here, we aimed to understand the cause of the short reproductive lifespan and hypothesized that SAC integrity was compromised. In comparing oocytes from young and sexually mature *Aurkb* cKO females, we found that SAC integrity becomes compromised rapidly with maternal age. We show that the increased desensitization of the SAC is driven by reduced expression of MAD2, ZW10 and Securin proteins, key contributors to the SAC response pathway. The reduced expression of these proteins is the result of altered protein homeostasis, likely caused by the accumulation of reactive oxygen species. Taken together, our results demonstrate a novel function for AURKB in preserving the female reproductive lifespan possibly by protecting oocytes from oxidative stress.

## Graphical abstract

AURKB in needed to preserve the female reproductive lifespan likely by protecting oocytes from oxidative stress. We show that Aurkb KO oocytes prematurely accumulate ROS during maternal aging. Prophase‐arrested oocytes from older females have reduced expression of SAC signaling components, causing a weakened SAC response during meiosis I. We propose the reduced expression of these proteins is the result of altered protein homeostasis, caused by the accumulation of ROS.

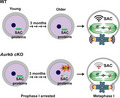

## 1 INTRODUCTION

In humans, aneuploidy in female gametes increases with maternal age and is the leading cause of early miscarriages (Gruhn et al., 2019; Nagaoka et al., 2012). Most cell types prevent aneuploidy by employing the spindle assembly checkpoint (SAC), a mechanism that integrates kinetochore–microtubule (K‐MT) occupancy with cell‐cycle progression (Musacchio, 2015). SAC activation occurs via a response to unoccupied kinetochores. Kinetochore‐localized MPS1 initiates the response and triggers recruitment of scaffold proteins that assist in assembling the mitotic checkpoint complex (MCC). The MCC, composed of MAD2, BUB3 and BUBR1, diffuses and sequesters CDC20, thereby preventing anaphase promoting complex/cyclosome (APC/C) activation and anaphase onset (Musacchio, 2015). In somatic cells, one unoccupied kinetochore is sufficient to elicit a SAC‐mediated cell‐cycle arrest (Kuhn & Dumont, 2019; Rieder et al., 1994). However, in mammalian oocytes, the SAC is more permissive, and can fail to prevent anaphase onset in the presence of several misaligned chromosomes (Gui & Homer, 2012; Kolano et al., 2012; Lane et al., 2012; Nagaoka et al., 2011; Sebestova et al., 2012). This permissiveness may prime oocytes to chromosome segregation errors in meiosis I (MI).

If SAC defects during MI can explain the high levels of aneuploidy in female meiosis, and, if it deteriorates with increasing maternal age, have therefore been long‐standing questions. Consistent with this hypothesis, oocytes from older humans and mice reportedly have reduced expression of SAC proteins (Nabti et al., 2017; Pan et al., 2008; Riris et al., 2014; Steuerwald et al., 2001). Whether this reduction in expression affects the integrity of the SAC is not known. However, oocytes from aged mice can arrest at metaphase I (Met I) when all kinetochores are unoccupied, suggesting that the SAC is not ablated by maternal age (Duncan et al., 2009). Therefore, further studies are needed to understand the contribution of SAC dysfunction to the maternal age effect on egg quality.

In somatic cells, Aurora kinase B (AURKB) activates the SAC through triggering depolymerization of microtubules that are improperly bound to kinetochores (DeLuca et al., 2006) and through the recruitment of SAC proteins to kinetochores (Santaguida et al., 2011). Mouse oocytes express an additional Aurora kinase, AURKC, which functionally replaces AURKB. The relative contributions of AURKB and/or AURKC to SAC function in oocytes remains unknown and have been challenging to resolve due to their highly similar sequences and behaviors. To untangle the roles of these kinases in the SAC in mouse oocytes, we used a genomic approach, harnessing knockout (KO) strains. Previous evaluation of these KO models revealed that *Aurkc* KO mice have normal reproductive lifespans and produce euploid metaphase II‐arrested eggs. In contrast, mice that lack *Aurkb* specifically in their oocytes (*Aurkb* cKO) undergo premature age‐related infertility, beginning as early as 3 months of age. As the animals aged, there was a decrease in litter size and an accompanied increase in aneuploid egg production (Nguyen et al., 2018). The similarity of these *Aurkb* cKO phenotypes with phenotypes that arise when there are SAC defects led us to hypothesize a specific role for AURKB in ensuring SAC integrity in preventing age‐related reproductive decline.

Here, we find that *Aurkb* cKO oocytes cannot generate an efficient SAC response in MI, which can increase the incidence of aneuploid eggs. These phenotypes are specific to the loss of AURKB because oocytes from *Aurkc* KO females arrest in response to unattached kinetochores, suggesting a specific requirement for AURKB in maintaining SAC integrity. Importantly, we show that the SAC defect observed in *Aurkb* cKO oocytes can be explained by an age‐related reduction in the expression of key SAC proteins. Because oxidative stress (OS), another hallmark of maternal gamete aging (Tarín, 1995), can alter protein homeostasis (Ghosh & Shcherbik, 2020; Stadtman & Levine, 2000), we evaluated reactive oxygen species (ROS) accumulation in cKO oocytes. We observed premature accumulation of ROS in older *Aurkb* cKO prophase I‐arrested oocytes when compared to oocytes from wild‐type (WT) animals, consistent with an alteration in protein homeostasis. This change in protein homeostasis leads to altered expression of critical SAC proteins. We conclude that AURKB is a key player in regulating reproductive longevity in females.

## 2 RESULTS

### 2.1 Increased aneuploidy is age dependent in *Aurkb* KO eggs

We previously showed that *Aurkb* cKO female fertility rapidly declines with increasing maternal age. Although *Aurkb* cKO females initially produce litter sizes comparable to WT, at ~3 months of age litter sizes progressively start to decline, with eventual loss of fertility by 5 months. Consistent with the fertility decline, we showed that a significant proportion of eggs from *Aurkb* cKO females are aneuploid, many containing prematurely separated sister chromatids (PSSC) (Nguyen et al., 2018).

**FIGURE 1:**
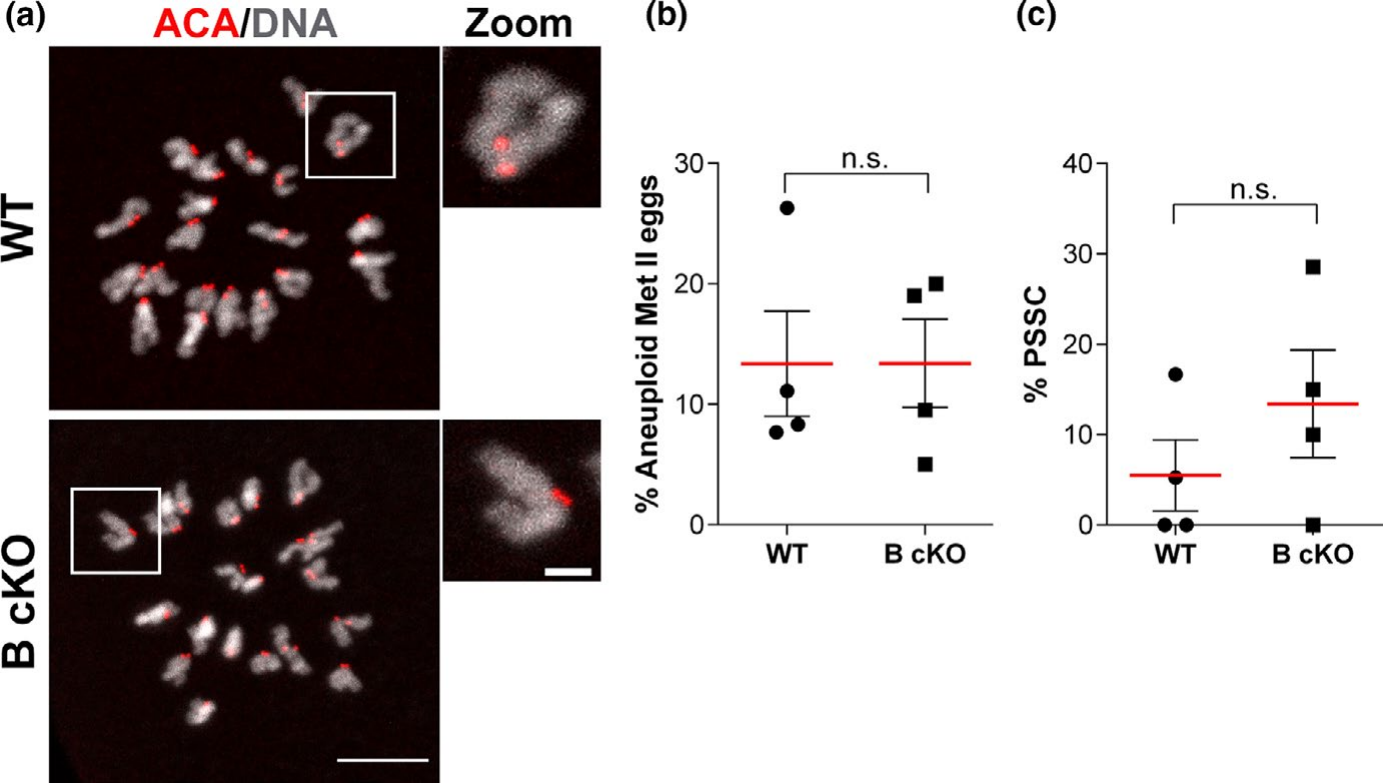
*Aurkb* cKO eggs from young females do not have elevated aneuploidy levels. (a) Representative confocal images of *in situ* chromosome spreads of Metaphase II (Met II) eggs from young females wild‐type (WT) or conditional *Aurkb* knockouts (B cKO) immunostained with anti‐centromeric antigen (ACA) (red) and DAPI to detect sister chromatids (gray). Examples of an individual pair of sister chromatids shown in the zoom. (b) Quantification of % of aneuploid Met II eggs (Unpaired Students t‐Test, two‐tailed, *p*=0.9960; number of oocytes WT: 62, B cKO: 75; 4 mice/genotype). (c) Quantification of the % of eggs with premature separated sister chromatids (PSSC) (Unpaired Students t‐Test, two‐tailed, *p*=0.3094; number of oocytes WT: 62, B cKO: 75; 4 mice/genotype). Graphs show individual animal values with the mean ±SEM from the 4 experiments. n.s.: not significant. Scale bars: 10 μm and 2 μm

The presence of PSSC and aneuploidy are hallmarks of female reproductive aging (Gruhn et al., 2019; Zielinska et al., 2019). Because the fertility of young *Aurkb* cKO females was normal (Nguyen et al., 2018), we hypothesized that the increased frequency of aneuploidy in *Aurkb* cKO eggs is age dependent. To test this hypothesis, we evaluated the number of chromosomes in WT and *Aurkb* cKO eggs from young females (1‐month‐old) by performing in situ chromosome spreads. In contrast to aneuploidy rates from older animals (~3 months) described previously (Nguyen et al., 2018), young *Aurkb* cKO animals generated euploid eggs at rates comparable to age‐matched WT females (Figure 1a, b). Consistent with normal chromosome numbers, sister chromatids remained associated with one another (Figure 1c). These data, combined with our published findings, suggest that AURKB is required for the maintenance of egg euploidy as maternal age increases.

### 2.2 SAC integrity weakens with age in *Aurkb* cKO females

**FIGURE 2:**
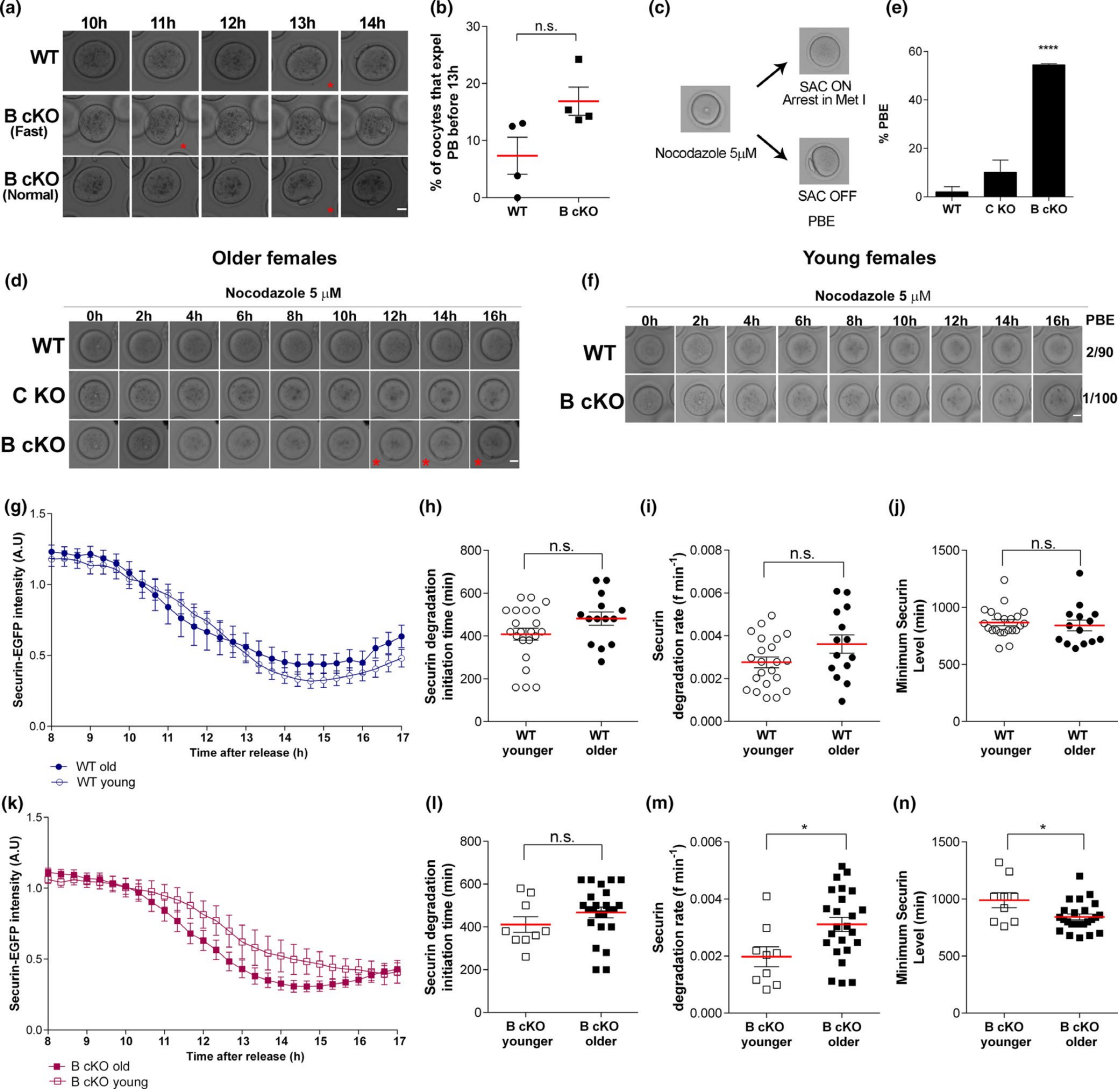
The SAC integrity in *Aurkb* cKO oocytes from older females is weak. (a) Representative images of timing of polar body (PB) extrusion in oocytes from wild‐type (WT) or conditional *Aurkb* knockouts (B cKO) from older females. Scale bar: 20 μm (b) Quantification of the % of oocytes that undergo PB extrusion before 13 h (Unpaired Students t‐Test, two‐tailed, *p *= 0.0576). Oocytes from 4 mice/genotype were examined and graphed independently. (c) Experimental design to evaluate SAC integrity. (d) Representative images of timing of PBE of oocytes from older females, matured in nocodazole. Red star indicates a PB. Scale bar: 20 μm. (e) Quantification of % of oocytes from (d) of the indicated genotypes that undergo PBE in nocodazole. C KO =Aurkc knockout. Oocytes from 3 WT, 3 C KO and 4 B cKO mice were examined (One‐way ANOVA, ****p < 0.0001). (f) Representative images of PBE timing of oocytes from young females, matured in nocodazole. Numbers of oocytes that expelled a PB relative to total number of oocytes analyzed is at the right of the images. Oocytes from 4 mice/genotype were examined. Scale bar: 20 μm. (g, k) Graph showing the mean degradation profiles of Securin‐gfp in oocytes from younger (open symbols), and older (closed symbols) WT (g) and *Aurkb* cKO (k) animals. (h, l) Quantification of initiation time for Securin degradation in WT (h) (*p *= 0.0976) and *Aurkb* cKO (l) oocytes (*p *= 0.2367). (i, m) Securin degradation rate in WT (i) (*p *= 0.0747) and *Aurkb* cKO (m) oocytes (*p *= 0.0188). (j, n) Time for the minimum level of securin in WT (j) (*p *= 0.6169) and *Aurkb* cKO (n) oocytes (*p *= 0.0171). Number of oocytes examined, WT young: 22, WT older: 14, B cKO young: 9, B cKO older; 24; 3 mice/genotype/age. n.s.: not significant. Graphs show individual oocytes values plus the mean ±SEM from 3 experiments. Analyses g‐n were Unpaired Students t‐Test, two‐tailed

Aneuploidy in metaphase II‐arrested eggs originates from chromosome segregation errors in MI. To determine the source of these errors in oocytes from older *Aurkb* cKO animals, we first assessed the timing of meiotic maturation by quantifying the time it takes oocytes to complete MI in vitro by monitoring polar body extrusion (PBE). The average time it took WT oocytes to extrude a polar body (PB) was 13h. We observed a trend that some oocytes from *Aurkb* cKO mice expelled PBs more rapidly (1‐2h) compared to WT (Figure 2a, b), and this contrasts with *Aurkc* KO oocytes that undergo PBE with slower kinetics (Schindler et al., 2012). Importantly, these differences in meiotic maturation timing highlight MI functions specific to AURKB.

Increased cell‐cycle kinetics are a hallmark of defective SAC signaling (Homer et al., 2005). In mitosis, AURKB is important for SAC function, however, whether this role exists in meiosis, or whether both AURKB/C are required remain unknown (Vallot et al., 2018). To determine if a dysfunctional SAC underlies the elevated aneuploidy levels observed in *Aurkb* cKOs, we isolated oocytes from older females and challenged SAC integrity by culturing them in nocodazole (Figure 2c). Oocytes cultured in nocodazole fail to establish kinetochore–microtubule attachments, and, if the SAC is functioning properly, oocytes will arrest at Metaphase of MI (Met I). However, if the SAC is weak or not functional, oocytes fail to arrest the cell cycle and extrude a PB. When cultured in nocodazole, 2% and 10% of WT and *Aurkc* KO oocytes, respectively, extruded a PB, indicating a functioning SAC. In contrast, 50% of oocytes from *Aurkb* cKO females extruded a PB, indicating a dysfunctional SAC response (Figure 2d, e). Furthermore, to test the hypothesis that the SAC becomes weak with age, we next evaluated whether a similar SAC dysfunction was present in oocytes from young *Aurkb* cKOs. In contrast to oocytes from older *Aurkb* cKOs, nearly 100% of oocytes from young females arrested at Met I when cultured in nocodazole, indicating a functional SAC (Figure 2f). These data suggest that AURKB, but not AURKC, maintains SAC integrity during maternal aging in oocytes.

Another marker used to measure SAC strength is the APC/C‐dependent turnover of Securin, the protein that prevents Separase from cleaving cohesin until SAC satisfaction. In oocytes from WT mice that are over 1 year old, Securin turnover is accelerated compared to oocytes from young animals (Nabti et al., 2017). We therefore reasoned that Securin turnover rates would be accelerated in *Aurkb* cKO oocytes from 3‐month‐old females compared to 1‐month‐old. We expressed EGfp‐tagged Securin in young and old WT and *Aurkb* cKO oocytes and monitored its turnover by live‐cell imaging. In oocytes from WT females, regardless of age, Securin‐EGFP signals started to decline at a similar time, decreased with comparable kinetics, and were reduced to the same level (Figure 2g‐j). Conversely, Securin‐EGFP degradation occurred more rapidly, with an overall greater level of degradation observed in oocytes from older females compared to oocytes from younger females (Figure 2k‐n).

**FIGURE 3:**
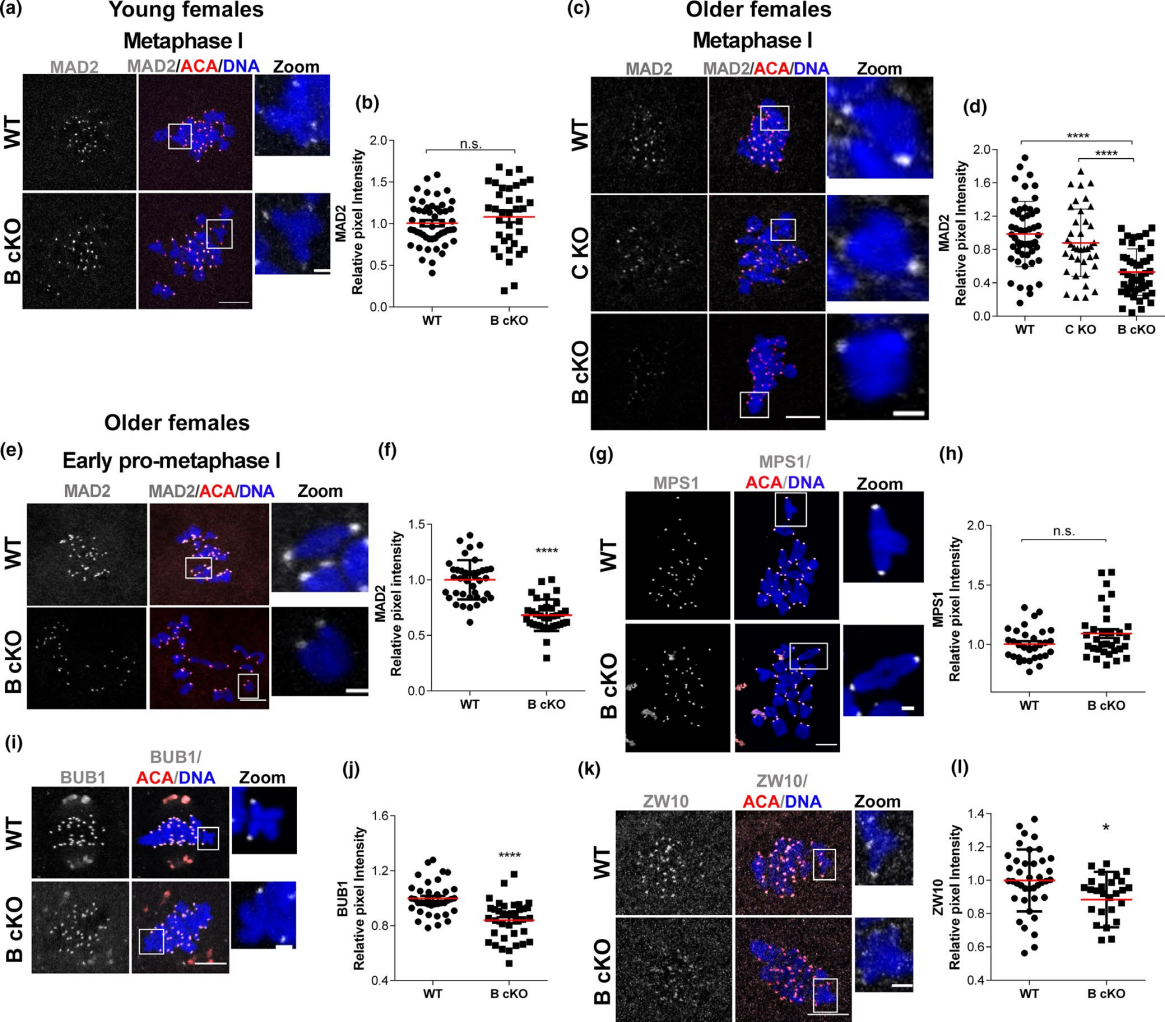
SAC mediator and scaffolding proteins are reduced at kinetochores in *Aurkb* cKO oocytes from older females. (a, c, e, g, I, k) Representative confocal images of oocytes from wild‐type (WT) or conditional *Aurkb* knockouts (B cKO) from young and older females. Centromeres were detected by staining with antibodies against ACA (red) and chromosomes were detected with DAPI‐staining (blue). (a, c) Representative confocal images of Met I oocytes immunostained with antibody against MAD2 (gray) from young (a) or older females (c). (b) Quantification of MAD2 intensity at kinetochores in (a) (*p *= 0.2711; number of oocytes examined, WT: 51, B cKO: 36; 4 mice/genotype). n.s.: not significant. (d) Quantification of MAD2 intensity at kinetochores in (c) (One‐way ANOVA, **** *p *< 0.0001; number of oocytes examined, WT: 52, B cKO: 47, C KO: 39; 3 mice/genotype). (e) Early pro‐metaphase I oocytes immunostained with antibodies against MAD2 (gray). (f) Quantification of MAD2 intensity at kinetochores in (e) (****p < 0.0001; number of oocytes examined, WT: 40, B cKO: 35; 6 mice/genotype). (g) Late pro‐metaphase I oocytes immunostained with anti‐MPS1 (gray); (h) Quantification of MPS1 intensity at kinetochores of (g) (*p *= 0.0514; number of oocytes examined, WT: 31, B cKO: 33; 3 mice/genotype). (i, k) Representative confocal images of metaphase I oocytes immunostained with (i) BUB1 (gray) or (k) ZW10 (gray). (j) Quantification of BUB1 intensity at kinetochores showed in (i) (**** *p *< 0.0001; number of oocytes examined, WT: 39, B cKO: 37; 3 mice/genotype). (l) Quantification of ZW10 intensity at kinetochores showed in (k) (* *p*<0.0121; number of oocytes examined, WT: 40, B cKO: 27; 3 mice/genotype). n.s.: not significant. Examples of individual bivalent (boxes) are magnified and shown in the zoom panels. Scale bars: 10 μm and 2 μm. Graph shows the mean value per oocyte of at least 30 kinetochores measured for each oocyte and includes the mean ±SEM from 3 experiments. Except for panels c‐d, Unpaired Students t‐Test, two‐tailed used

Activation of the SAC relies on the establishment of the MCC at unattached kinetochores. A core component of the MCC, MAD2, is commonly used as an indicator of SAC activity and strength (Wassmann et al., 2003). To determine whether AURKB is required for MCC localization and SAC activation, we evaluated the levels of MAD2 at kinetochores in WT and *Aurkb* cKO Met I oocytes from young and older females. Consistent with the ability to elicit a strong SAC‐induced arrest in the presence of unattached kinetochores (Figure 2f), oocytes from young animals exhibited similar levels of MAD2 at Met I kinetochores (Figure 3a, b). However, in oocytes from older *Aurkb* cKO females, kinetochore‐localized MAD2 was reduced by ~50% compared to levels in oocytes from WT and AURKC KO aged‐matched controls (Figure 3c, d). These data are consistent with aged *Aurkb* cKO oocytes failing to arrest in nocodazole (Figure 2d‐e), indicating a weakened SAC response. These data suggest a requirement for AURKB in the maintenance of SAC integrity in oocytes with age.

Because SAC activity responds to kinetochore occupancy, we next evaluated whether the weaker SAC response observed in older *Aurkb* cKO oocytes correlated with an increase in abnormalities in K‐MT attachments at Met I. Surprisingly, *Aurkb* cKO oocytes rarely had abnormal or unattached kinetochores (4%), similar to WT. On the other hand, *Aurkc* KO oocytes had a statistically significant increase in the number of abnormal K‐MT attachments (13.6%) (Figure S1a, b), suggesting that AURKC, and not AURKB, is required to destabilize abnormal K‐MT attachments during oocyte meiotic maturation. These findings suggest that the SAC defects in *Aurkb* cKO oocytes arise for reasons unrelated to the correction of abnormal K‐MT attachments.

### 2.3 BUB1/RZZ SAC scaffolding proteins are reduced in *Aurkb* cKO oocytes

We next sought to define the source for the decreased levels of MAD2 at kinetochores observed in *Aurkb* cKO oocytes from older females. Early pro‐metaphase I is a period of a significant recruitment of MAD2 to kinetochores. To determine if the decreased levels of MAD2 were the result of the inability to establish MAD2 at unattached kinetochores, we quantified MAD2 levels in pro‐metaphase I oocytes from older WT and *Aurkb* cKO females. In contrast to WT oocytes, oocytes from *Aurkb* cKOs had significantly reduced MAD2 at kinetochores (Figure 3e, f). This reduction was similar to that observed at Met I in *Aurkb* cKO oocytes, suggesting that AURKB is required for MCC establishment at kinetochores.

To further define the molecular mechanisms by which AURKB regulates SAC integrity, we evaluated the localization of additional components of the checkpoint signaling pathway. We first investigated the recruitment of MPS1 to meiotic kinetochores, because this kinase is the initial signaling molecule recruited to unoccupied kinetochores in the SAC signaling cascade (Musacchio, 2015). Surprisingly, kinetochore‐localized MPS1 levels were not significantly different in *Aurkb* cKO oocytes compared to WT, both in maturing oocytes and those arrested at Met I via nocodazole treatment (Figure 3g, h; S2a, b). These data indicate that AURKB functions downstream of the initial SAC response. Next, we determined if other scaffold proteins involved in MAD2 recruitment were perturbed at Met I. In mitosis, MAD2 recruitment depends on BUB1/BUB3 and the RZZ (Rodd/Zwilch/Zw10) complex (Rodriguez‐Rodriguez et al., 2018). We therefore quantified the levels of BUB1 and ZW10 at Met I kinetochores in oocytes from older *Aurkb* cKOs. Compared to oocytes from WT, *Aurkb* cKO oocytes had reduced kinetochore‐localized BUB1 (Figure 3i, j) and ZW10 (Figure 3k, l) suggesting that the recruitment of the scaffold component of the SAC pathway is altered in the absence of AURKB. These results are consistent with previous studies that found a reduction in MAD2 localization after depletion of ZW10 in mouse oocytes (Park et al., 2019).

Kinetochore structure is also critical for the recruitment of SAC proteins (Musacchio, 2015; Wynne & Funabiki, 2015). We therefore tested if the reduction in localization of the scaffold complex proteins was due to an alteration in kinetochore structure by evaluating levels of kinetochore‐localized HEC1, the most proximal domain of the kinetochore interface whose localization is dependent upon proper assembly of upstream kinetochore components (DeLuca et al., 2006). HEC1 levels at kinetochores in *Aurkb* cKO oocytes were similar to WT (Figure S3 a, b), suggesting that AURKB is not required for kinetochore assembly. Collectively, these results suggest that AURKB functions upstream of the BUB1/BUB3/RZZ scaffold to ensure sufficient MAD2 recruitment to kinetochores, critical for a robust SAC signaling cascade during MI.

### 2.4 Total SAC protein levels are reduced in *Aurkb* cKO oocytes in an age‐dependent manner

**FIGURE 4:**
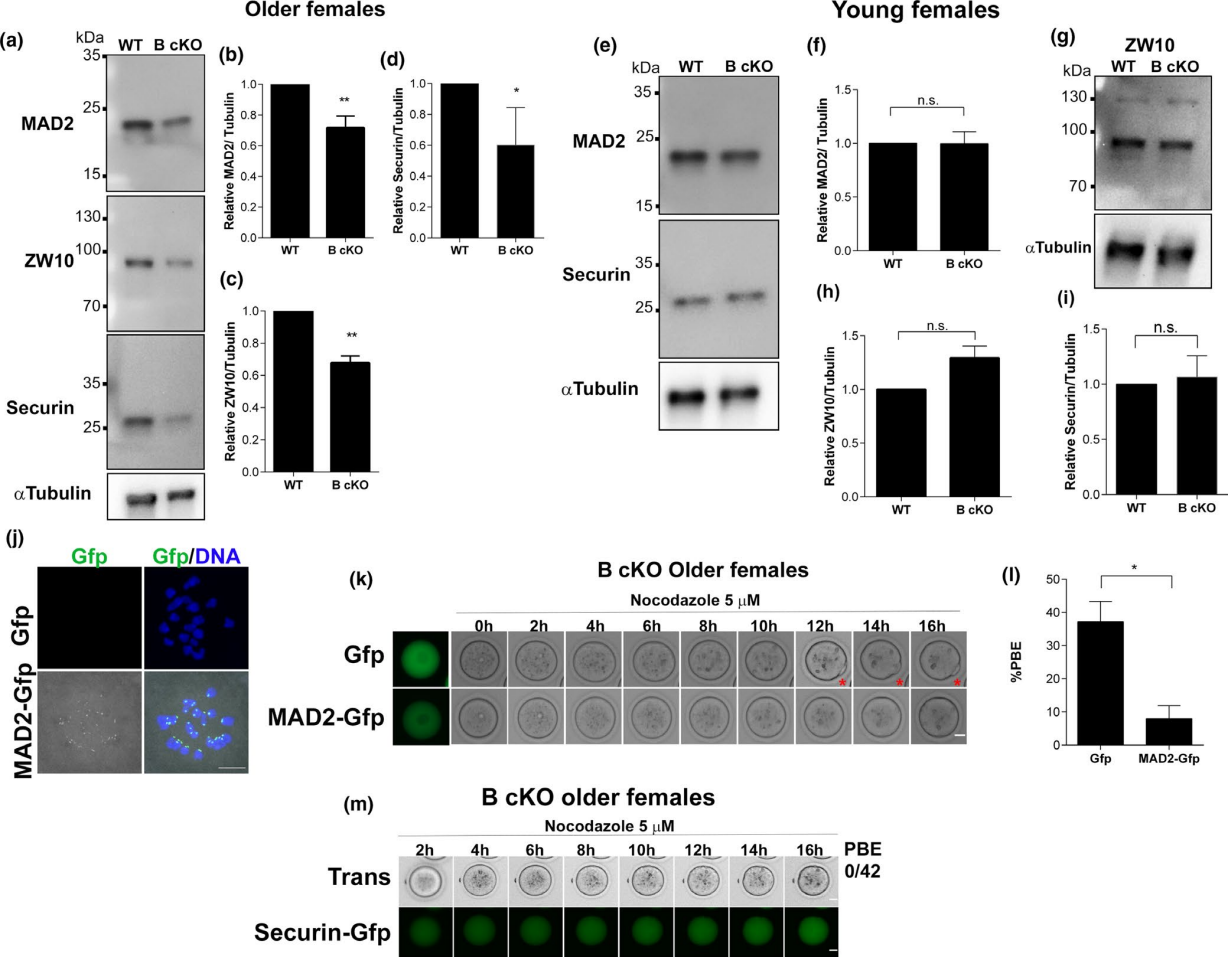
*Aurkb* cKO oocytes from older females have reduced expression of MAD2, ZW10 and Securin. (a) Representative Western blot images of prophase I‐arrested oocytes from older wild‐type (WT) or conditional *Aurkb* knockout (B cKO) females detecting MAD2, ZW10 and Securin. (b) Quantification of MAD2 (**p < 0.0030), (c) Quantification of ZW10 (** *p *< 0.0016). (d) Quantification of Securin (*p < 0.0468). (e, g) Representative Western blots of prophase I‐arrested oocytes from young females detecting MAD2, Securin (d) and ZW10 (g). (f) Quantification of MAD2 (*p *= 0.9774). Graph shows individual values plus the mean ±SEM from 3 independent experiments. (h) Quantification of ZW10 (*p *= 0.1166). (i) Quantification of Securin (*p < 0.7505), MAD2, ZW10 and Securin signals were normalized to α‐Tubulin. Each lane contained 100 prophase I‐arrested oocytes. n.s.: not significant. Graph shows mean ±SEM from 2–3 independent experiments. (j) Representative confocal images of pro‐metaphase I oocytes expressing Gfp or MAD2‐gfp (green) DNA (blue). Scale bar: 10 μm. (k) Representative images of timing of PBE of *Aurkb* cKO oocytes from older females, matured in nocodazole expressing either Gfp or MAD2‐gfp. Red star indicates a PB. Scale bar: 20 μm. (l) Quantification of % of oocytes from (k) that undergo PBE in nocodazole. (*p = 0.0.0160; number of oocytes examined Gfp: 73, MAD2‐gfp: 59; 6 mice). Graph shows the mean ±SEM from 3 experiments. (m) Representative images of timing of PBE of *Aurkb* cKO oocytes from older females, matured in nocodazole expressing Securin‐gfp (green). Numbers of oocytes that expelled a PB relative to total number of oocytes analyzed is indicated to the right of the images. Unpaired Students t‐Test, two‐tailed was used

Although *Aurkb* cKO oocytes have reduced levels of MAD2, BUB1 and ZW10 at kinetochores, we wanted to confirm that this defect was not due to changes in expression levels. We measured total protein levels in prophase I‐arrested oocytes by Western blotting. We note that we were unable to evaluate BUB1 due to antibody availability. Unexpectedly, MAD2, ZW10 and Securin protein levels were reduced by ~30% in *Aurkb* cKO oocytes from older animals (Figure 4a‐d), suggesting that AURKB regulates SAC activity in mouse oocytes directly by promoting the recruitment of MAD2 to kinetochores and/or indirectly by regulating protein levels. These results are consistent with a previous report that found a reduction in Securin expression in WT oocytes from 1‐year‐old women (Nabti et al., 2017). Next, we asked if the decline in SAC protein levels in *Aurkb* cKO females was age dependent. To answer this question, we evaluated these proteins from young females and found that the levels of these proteins were comparable to levels in WT oocytes (Figure 4e‐i).

Next, we reasoned that if MAD2 and Securin protein were limiting to produce and maintain a robust SAC response, then their overexpression could rescue the SAC defect. First, we confirmed that exogenous MAD2‐Gfp localized to kinetochores in WT oocytes at Met I (Figure 4j). Next, *Aurkb* cKO oocytes from older females were injected with *Gfp* or *Mad2*‐*Gfp* RNA and imaged live to monitor PBE while maturing in nocodazole. In *Gfp*‐injected *Aurkb* cKO oocytes, ~40% of oocytes failed to arrest and extruded PBs, as before (Figure 2e; Figure 4k, l). Importantly, when KO oocytes expressed *Mad2*‐*Gfp*, nearly all (~95%) the oocytes arrested at Met I and did not extrude PBs. Similar results were observed when *Aurkb* cKO oocytes from older females were injected with *Securin*‐*Gfp*: 100% of the oocytes arrested in Met I while maturing in nocodazole (Figure 4m). These data indicate that the decay in SAC integrity in *Aurkb* cKO oocytes is associated with an age‐related decline in the expression of SAC proteins.

### 2.5 ROS levels increase with age in *Aurkb* cKO oocytes

**FIGURE 5:**
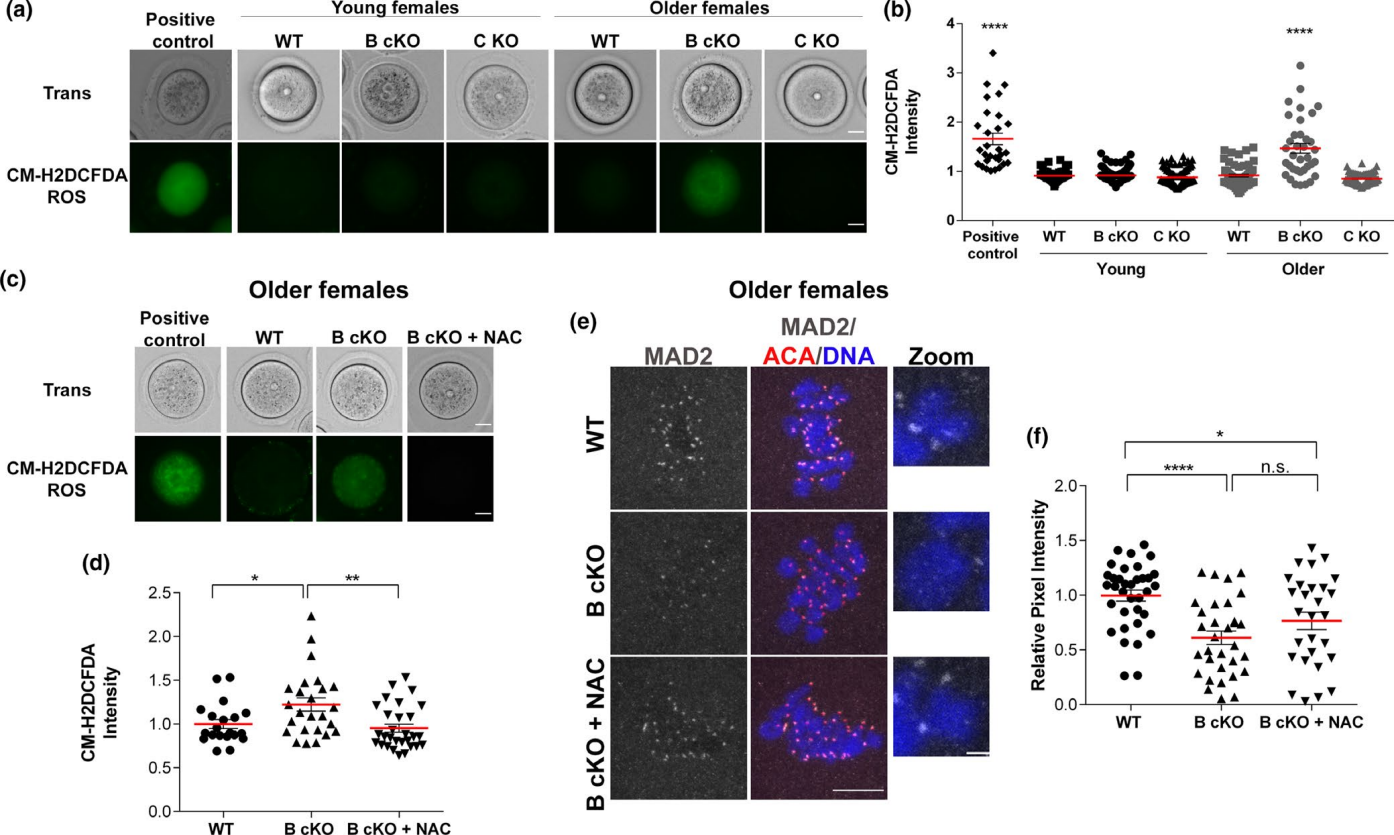
ROS levels are increased in *Aurkb* cKO oocytes from older females. (a) Representative images of CM‐H2DCFDA fluorescence in prophase I‐arrested oocytes from wild‐type (WT), conditional *Aurkb* knockouts (B cKO), or *Aurkc* knockout (C KO) females at different ages. (b) Quantification of CM‐H2DCFDA intensity showed in (a) ****p < 0.0001; number of oocytes examined: positive control: 29; young WT: 58, B cKO: 77, C KO: 92; older: WT: 60, B cKO: 36, C KO: 70; 3 mice/genotype). (c) Representative images of CM‐H2DCFDA fluorescence in prophase I‐arrested oocytes from wild‐type (WT), conditional *Aurkb* knockouts (B cKO), older females treated with and without NAC. Positive control: WT oocytes incubated with 200 μM H_2_O_2_. Scale bars: 20 μm. (d) Quantification of CM‐H2DCFDA intensity showed in (c) (**p = 0.0035; * *p *< 0.05; number of oocytes examined: WT: 20, B cKO: 25, B cKO +NAC:29; 3 mice/genotype). (e) Representative confocal images of WT and *Aurkb* cKO Met I oocytes from older females treated with or without NAC, immunostained with antibodies against ACA (red), MAD2 (gray) and DAPI (blue). (f) Quantification of MAD2 intensity at kinetochores in (e) (*p = 0.0464; **** *p *< 0.0001; n.s. not significant; number of oocytes examined: WT: 35, B cKO: 32, B cKO +NAC:28; 3 mice/genotype). Graph shows individual oocyte values plus the mean ±SEM from 3 experiments. One‐way ANOVA used

To determine what pathways could explain the reduction in SAC protein expression levels and aging phenotypes observed in *Aurk*b cKO oocytes, we explored possible connections reported in the literature. A previous study documented a decrease in localized AURKB and MAD2 in zygotes upon H_2_O_2_‐induced OS, and that MAD2 localization depends upon AURKB activity (Li et al., 2019). Increased OS is associated with maternal reproductive aging (Tarín, 1995), and with changes in protein levels (Ghosh & Shcherbik, 2020; Topf et al., 2018). We hypothesized that oocytes from older *Aurkb* cKO females more rapidly accumulate ROS compared to WT. To test this hypothesis, we compared ROS levels in WT and *Aurkb* cKO prophase I‐arrested oocytes from young and older females. *Aurkc* KO oocytes were included as an additional control. ROS levels in oocytes from young animals were low and did not differ between any of the genotypes (Figure 5a, b). In contrast, whereas ROS levels remained low in WT and *Aurkc* KO oocytes from older animals, it significantly increased in *Aurkb* cKO oocytes (Figure 5a, b).

We next sought to determine if the increase in ROS observed in aged *Aurkb cKO* oocytes could be responsible for the decrease in kinetochore‐localized SAC proteins. We incubated prophase I‐arrested oocytes from older females with an antioxidant (N‐Acetyl‐L‐Cysteine (NAC)) for 14h prior to maturation to Met I. Incubation in NAC reduced ROS levels in oocytes from older *Aurkb cKO* females (Figure 5c, d), but MAD2 levels were not restored at kinetochores, remaining significantly lower than WT (Figure 5e, f). In our genetic system, *Aurkb* is deleted early in oogenesis, months before the acute NAC exposure. Because protein levels were not restored, the data suggest that the ROS impact on SAC protein levels occurs during oocyte growth. Future work will be needed to untangle the precise mechanisms by which AURKB regulates ROS accumulation in aging.

**FIGURE 6:**
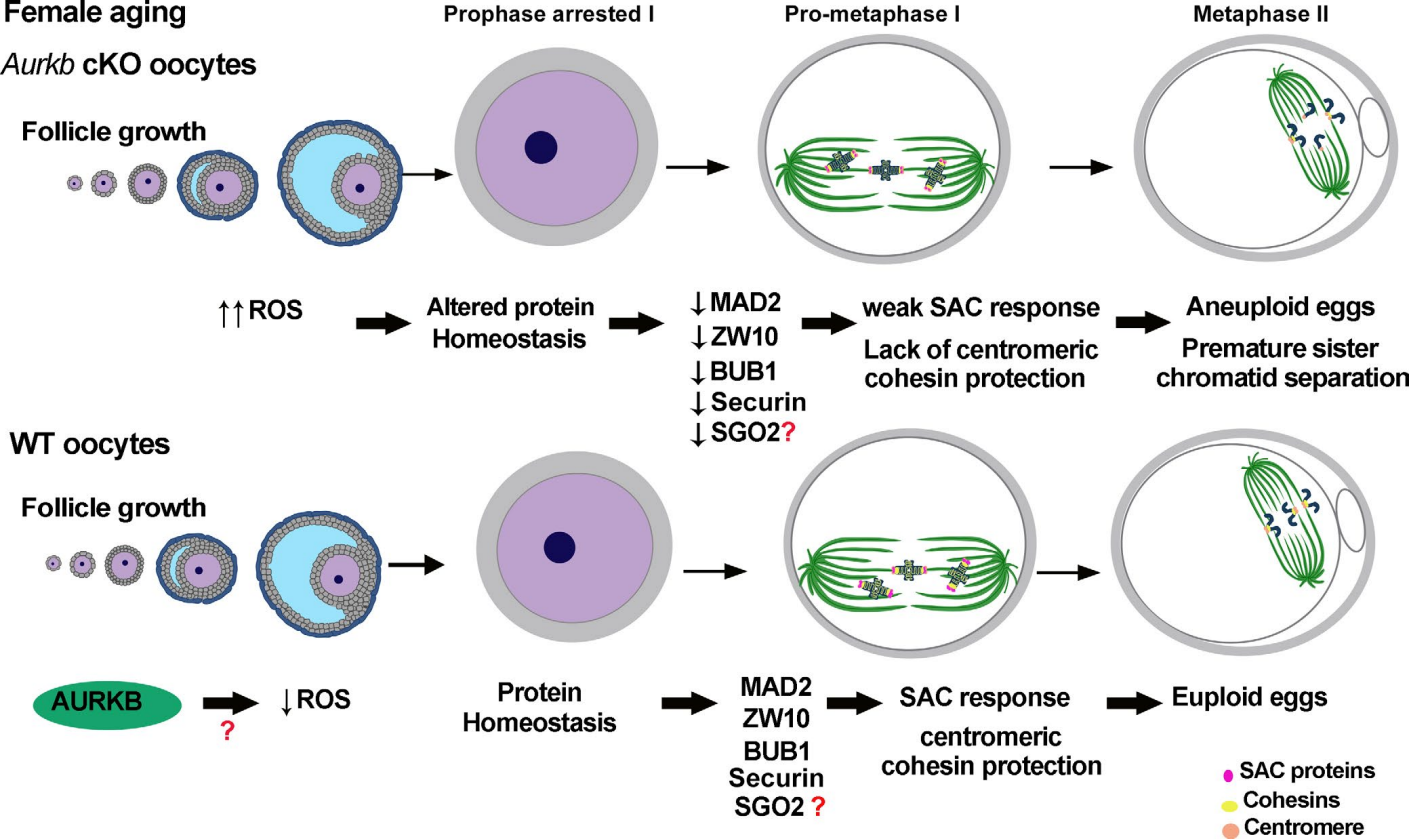
Model of the role of AURKB in preventing premature aging in mouse oocytes. Schematic summarizing our findings for a requirement of AURKB in protecting aging oocytes from ROS‐induced changes of protein expression that weaken the SAC. This may or may not be a direct role

Our data support a model in which changes in protein homeostasis, possibly from ROS accumulation, drive an age‐related decrease in SAC integrity in *Aurkb* cKO oocytes (Figure 6). We show that ROS more rapidly accumulates in the absence of AURKB, likely perturbing translation or other processes that contribute to proteome stability. This alteration reduces the amount of critical SAC components that can be recruited to kinetochores and therefore compromises the integrity of the SAC. Over time, these defects worsen, increasing aneuploidy rates and causing infertility. Therefore, we propose that AURKB is key to protecting reproductive longevity in females.

## 3 DISCUSSION

Maternal age is a main cause of fertility decline (Gruhn et al., 2019; Wang et al., 2020), yet the underlying mechanisms that contribute to declining egg quality with increasing maternal age are still not completely understood. *Aurkb* cKO female mice display characteristic outcomes of premature reproductive aging: PSSC, aneuploidy and a rapid decline in fertility (Nguyen et al., 2018). Here, we aimed to understand why *Aurkb* cKO females have premature age‐related infertility. We compared oocytes from *Aurkb* cKO young females (1 month) to older cKO females (3 months). We demonstrate that *Aurkb* cKO oocytes have an age‐dependent decline in SAC integrity which can explain our previous findings of elevated aneuploidy. This decline occurs concomitantly with a reduction in the expression of SAC scaffold and mediator proteins in prophase I‐arrested oocytes. We speculate that the result of this decline is an inability to recruit enough SAC pathway proteins to kinetochores resulting in a weakened SAC response. Moreover, we show that prophase I‐arrested oocytes from older *Aurkb* cKO females have higher levels of ROS than oocytes from younger animals. We propose that this increase in ROS perturbs protein homeostasis and therefore reduces the expression of SAC proteins, but a direct connection must still be tested. These data suggest that AURKB is involved in aging to maintain, regulate or sense the levels of OS and to avoid errors in MI chromosome segregation (Figure 6).

Studies that evaluate effects of maternal age in egg quality typically use mice ranging from 12–18 months. In this study, we show that when AURKB is deleted, oocytes present advanced aged phenotypes by 3 months, suggesting a crucial AURKB role in maintaining egg quality during maternal aging. Consistent with this model, the expression of *Aurkb* is upregulated in oocytes and eggs from older female mice and macaques, suggesting a protective function (Pan et al., 2008; Wang et al., 2020). Furthermore, in humans, a rare *AURKB* variant (c116T>C) is associated with improved fertility outcomes (Nguyen et al., 2017) consistent with AURKB expression and activity providing an advantage during human maternal aging.

Maternal aging is associated with OS (Tarín, 1995), increased mitochondrial dysfunction (May‐Panloup et al., 2016) and decreased expression of oxidative phosphorylation (Grøndahl et al., 2010; Zhang et al., 2019) and antioxidant genes (Wang et al., 2020; Zhang et al., 2019). Here, we show that *Aurkb* cKO oocytes from relatively young female mice have high levels of ROS and have a weak SAC because total protein levels of some SAC components are reduced. If the higher ROS levels directly affect SAC integrity is not well understood. OS is involved in regulating protein levels in many age‐associated diseases, by at least two different mechanisms. OS can cause oxidation of proteins inducing loss of function or degradation (Stadtman & Levine, 2000) or can affect the translational machinery, reducing protein synthesis levels (Ghosh & Shcherbik, 2020; Topf et al., 2018). In somatic cells, OS can override the SAC by degrading Securin and Cyclin B. Consistent with this role, we show that *Aurkb cKO* oocytes from older females have reduced Securin levels (Figure 4a, d) and have a faster Securin turnover rate (Figure 2k, m). Future studies to evaluate protein levels during meiotic maturation are needed to elucidate the molecular mechanism by which OS regulates protein homeostasis.

Because *Aurkb* cKO prophase I‐arrested oocytes from older females have higher ROS compared to age‐matched WT and to younger *Aurkb* cKO females, we propose that AURKB prevents ROS accumulation in mouse oocytes (Figure 6). In our conditional KO system, *Aurkb* is excised in primordial follicles, the follicle type that comprises the ovarian reserve (Lan et al., 2004); therefore, ROS accumulation likely occurs during follicle growth within the ovary of these KO females (Figure 6). Expression of antioxidant genes are downregulated in primary follicles during aging in non‐human primates (Wang et al., 2020). Moreover, in ovaries from aged females, secondary and antral follicles show secondary products of OS like 4‐hydroxynonenal and DNA damage (Lim & Luderer, 2011; Mihalas et al., 2017). Future studies to determine when ROS accumulates in *Aurkb* cKO oocytes will be informative in pinpointing this AURKB requirement. Consistent with premature accumulation of ROS during follicle growth, when *Aurkb* cKO prophase I‐arrested oocytes were treated with antioxidant, kinetochore‐localized MAD2 levels were not restored, despite rescue of ROS levels (Figure 5c‐f). If longer exposure of early‐stage follicles to antioxidants can rescue protein levels and SAC integrity should be further evaluated.

How AURKB is involved in ROS control or response is not known. One possibility involves the interplay between the Aurora kinase homologs. We previously described that in *Aurkb* cKO oocytes the activities of AURKA and AURKC are increased (Nguyen et al., 2018). In several cancer cell lines, AURKA reportedly has several non‐mitotic functions (Bertolin & Tramier, 2020). For example, AURKA localizes to the mitochondria matrix and regulates its morphology and dynamics (Bertolin et al., 2018), and overexpression of AURKA in these cells induces mitochondria elongation causing an increase in ATP production (Bertolin et al., 2021; Grant et al., 2018). We speculate that AURKB negatively regulates the activity of AURKA to indirectly protect oocytes from ROS accumulation. Further studies are needed to evaluate the role of AURKA in mitochondrial function in mouse oocytes. Alternatively, AURKB may have a direct role in responding to OS. OS causes DNA damage (Zhang et al., 2016) which, in mouse oocytes, triggers the SAC response and halts meiotic cell‐cycle progression (Collins et al., 2015). Moreover, in mouse embryos exposed to OS, AURKB ensures proper levels of MAD2 at kinetochores (Li et al., 2019), which is consistent with our findings. Taken together, these data suggest that AURKB is critical to ensure a SAC response to protect genome integrity.

## 4 CONCLUSION

We demonstrate that AURKB is crucial to maintain the reproductive lifespan in females possibly by protecting oocytes from premature ROS accumulation. This protection may promote protein homeostasis thereby ensuring that necessary levels of SAC signaling proteins are recruited to kinetochores to avoid aneuploidy. An exploration of AURKB expression levels and function during the maternal aging process should shed light on its utility as a biomarker of egg quality and/or how it can be used to improve reproductive outcomes.

## 5 MATERIALS AND METHODS

### 5.1 Mouse strains and genotyping

*Aurkc* ^−/−^ (C KO) mice, floxed *Aurkb* mice, and *Aurkb* ^fl/fl^ Gdf9‐Cre (B cKO) mice were described previously (Fernández‐Miranda et al., 2011; Lan et al., 2004; Nguyen et al., 2018; Schindler et al., 2012). Control animals (Wild‐type (WT)) are from the same genetic background but lack the Cre recombinase transgene. All animals were in a mixed background of C57BL/6J, 129/Sv, and CD1 and maintained following the Rutgers University Institutional Animal Use and Care Committee and National Institutes of Health guidelines. Mice were housed in 12–12 h light‐dark cycle, with constant temperature and with food and water provided ad libitum. All animal experiments performed in this study were approved by the Rutgers IACUC. All oocyte experiments were conducted using healthy female mice ranging in age from 3–12 weeks, and as specified in each experiment. Genotyping was performed prior to weaning and repeated upon use of the animals for experiments for replication and confirmation as previously described (Nguyen et al., 2018).

### 5.2 Oocyte isolation and in vitro maturation

Prophase I‐arrested oocytes were collected as described (Blengini & Schindler, 2018). 48 h prior collection, females were injected intraperitoneally with 5 I.U. of pregnant mare serum gonadotropin (PMSG) (Lee Biosolutions #493‐10). Ovaries were minced in bicarbonate free minimal essential medium (MEM) (Sigma #M0268) containing, 25 mM HEPES, pH 7.3, 3 mg/ml poyvinylpyrrolidone (PVP) containing 2.5 μM milrinone (Sigma‐Aldrich #M4659). Fully grown oocytes were matured in Chatot, Ziomek, and Bavister (CZB) media without milrinone in a humified incubator programmed to 5% CO_2_ and 37℃ for different periods of time, depending on which meiotic stage to be studied: 3 h (early pro‐metaphase I); 5 h (late pro‐metaphase I); 7.5 h (metaphase I).

### 5.3 Cold stable treatment and K‐MT attachment evaluation

To evaluate K‐MT attachments oocytes matured to Met I were incubated in cold MEM medium for 8 minutes following by immediate fixation. Oocytes were fixed in PBS containing 2% paraformaldehyde (PFA) +0.1% Triton X‐100 (Sigma‐Aldrich #900‐93–1) for 20 minutes and immunostained with anti‐centromeric antigens (ACA) and α–Tubulin, to visualize kinetochores and microtubules, respectively (Blengini & Schindler, 2018).

### 5.4 Live cell imaging

To evaluate the timing of PBE, prophase I‐arrested oocytes were matured *in vitro* for 24 h using an EVOS FL Auto Imaging System (Life Technologies) with a 10× objective. The microscope stage was heated to 37℃ and 5% CO_2_ was maintained using the EVOS Onstage Incubator. Images on bright field of individual cells were acquired every 20 min and processed using NIH Image J Software.

### 5.5 Immunocytochemistry

Immunofluorescence of fully grown oocytes was performed as before (Blengini & Schindler, 2018). Briefly, oocytes at the desired meiotic staged were fixed in PFA at room temperature (MAD2, ZW10: PFA (Sigma‐Aldrich #P6148), 2% in PHEM for 20 min, HEC1, BUB1: 2% PFA in PBS +0.1% Triton X‐100 for 20 min). Prior to immunostaining, oocytes were permeabilized in PBS containing 0.2% Triton for 20 min and blocked in blocking buffer (0.3% BSA containing 0.01% Tween in PBS) for 10 min. Immunostaining was performed by incubating in primary antibody for 1‐2 h in a dark, humidified chamber, followed by three washes of 10 min each in blocking solution. Then oocytes were incubated in secondary antibody for 1h in a dark humidified chamber, followed by three washes of 10 min each in blocking solution. After washing, oocytes were mounted in 5 μl of Vectashield containing 4, 6‐Diamidino‐2‐Phenylindole, Dihydrochloride (DAPI) (Life Technologies #D1306).

### 5.6 Chromosome spreads

Oocytes were matured *in vitro* until late pro‐metaphase I and were treated with Acidic Tyrode's solution (Millipore Sigma #MR‐004‐D) to remove the zona pellucida. Then, groups of 7–8 oocytes were transferred to a drop of chromosome spread solution (0.16% Triton‐X‐100, 3 mM DTT (Sigma‐Aldrich #43815), 0.64 PFA in distilled water) on glass slides and allowed to slowly air dry prior to processing for immunofluorescence. Immunostaining of chromosome spreads was performed by washing the slide two times with PBS for 10 min and blocking the slide in PBS supplemented with BSA 3% for 10 min. Primary antibody, to detect MPS1 and ACA, was incubated for 3 h in a dark, humidified chamber at room temperature, followed by three washes in PBS of 10 min each. Secondary antibody was incubated for 1.5 h in a dark, humidified chamber at room temperature followed by three washes in PBS of 10 minutes each. After washing, the slides were mounted in Vectashield containing DAPI (Life Technologies #D1306).

### 5.7 In situ chromosome counting

Prophase I‐arrested oocytes were collected from 4‐week‐old mice and matured in CZB media without milrinone in a humified incubator (5% CO_2_ and 37℃) for 14 h until they arrest at metaphase II. Then, eggs were cultured for 2 h in 100 µM Monastrol (Sigma #M8515) to collapse the spindle and separate the chromosomes. Finally, the eggs were fixed in 2% PFA in PBS for 20 min and permeabilized in PBS containing 0.2% Triton X‐100 for 20 min. Eggs were stained with ACA antibody to detect centromeres and DAPI to detect DNA. Normal chromosome counting for a mouse egg is 20 pairs of sister chromatids; any deviation of this number was considered an aneuploid egg. Chromosome counting was performed with NIH Image J software using cell counter plugins.

### 5.8 Microinjection

Prophase I‐arrested oocytes were microinjected in MEM medium with 100 ng/μl Securin‐Gfp and 300 ng/μl Mad2‐Gfp mRNAs. Microinjected oocytes were cultured for 3h in CZB medium supplemented with Milrinone to allow protein expression prior to experimental procedures.

### 5.9 Plasmids

Securin‐Gfp plasmid was previously described by (Solc et al., 2015). Human MAD2 was amplified by PCR and cloned into the pIVT–Gfp vector (Addgene #16047). This plasmid was linearized and the cRNA was prepared using an mMessage mMachine T7 kit (Ambion), according to manufacturer's protocol. The synthesized cRNAs were then purified using an RNAeasy kit (Qiagen) and stored at −80℃.

### 5.10 Intracellular ROS determination

Prophase I‐arrested oocytes were incubated for 30 min under oil at 37°C in CH‐H2DCFDA (ThermoFisher #C6827) (5 μM) diluted in CZB media supplemented with milrinone. Oocytes were washed three times and immediately imaged. For positive control, WT oocytes were incubated for 1 h in 200 μM H_2_O_2_ (Fisher Chemical # H323‐500). Images were acquired using an EVOS FL Auto Imaging System (Life Technologies) with a 20× objective. Oocytes were incubated with 5 mM N‐Acetyl‐L‐Cysteine (NAC) (Abcam #Ab143032), for 14h before milrinone release and then matured to Met I.

### 5.11 Western blotting

A total of 100 oocytes were mixed with SDS sample buffer (1% SDS, 1% β‐mercaptoethanol, 20% glycerol, 50 mM Tris‐HCl (pH 6.8) and the phosphatase inhibitors sodium fluoride (25 mM) and sodium orthovanadate (1 mM) and denatured at 95℃ for 5 minutes. Proteins were separated by electrophoresis in 10% SDS polyacrylamide precast gel (Bio‐Rad, #4561036). The separated proteins were transferred to nitrocellulose membranes (Bio‐Rad, #1704156) using a Trans‐Blot Turbo Transfer System (Bio‐Rad) and then blocked with 2% ECL blocking (Amersham, #RPN418) solution in TBS‐T (Tris‐buffered saline with 0.1% Tween 20) for 1h. The membranes were incubated overnight at 4°C with primary antibody to detect MAD2 (1:500) and ZW10 (1:500), Securin (1:500) or for 1 h to detect α‐Tubulin (1:500). The membranes were incubated with secondary antibody (1:1000; Kindle Bioscience #R1006) for 1 h at room temperature. The signals were detected using ECL Select western blotting detection reagents (Kindle Biosciences, KwikQuant Western Blot Detection Kit) following the manufacturer's protocol. Images were analyzed using Image J software (NIH) (Schneider et al., 2012) and were normalized to α‐Tubulin and set to 1 in WT.

### 5.12 Antibodies and drugs

The following antibodies were used for immunoblot (IB) and immunofluorescence (IF) experiments: Human anti‐ACA (1:30, Antibodies Incorporated #15‐234), Mouse anti α‐Tubulin Alexa Fluor 488 conjugated (IF:1:100, Life Technologies #322588), Rabbit anti‐ MAD2 (IF:1:1000, IB: 1:500; Biolegend #924601 or previously Covance #PRB‐452C)), sheep anti‐BUB1 (1:100, gift from Dr. S. Taylor), rabbit anti‐MPS1 (1:100, gift from Dr. H. Yu), rabbit anti‐Securin (IB: 1:500, Invitrogen #700791) rabbit anti‐ZW10 (IF:1:100; IB:1:500, Abcam #ab21582), rabbit anti‐HEC1 (1:100; gift from Dr. R. Benezra), rabbit anti α‐Tubulin WB:1:500 Cell Signaling Technology #11H10). The following secondary antibodies were used at 1:200 for IF experiments: goat‐anti‐human‐Alexa‐633 (Life Technologies #A21091), donkey‐anti‐rabbit‐Alexa‐568 (Life Technologies #A10042) and Cy5 secondary antibody pre‐absorbed against goat serum proteins (Jackson Immunoresearch). Monastrol (Sigma #M8515), and nocodazole (Sigma #M1404) were dissolved in dimethyl sulfoxide (DMSO) (Sigma) and added to the CZB culture media at final concentrations of 100 μM and 5 μM, respectively. N‐Acetyl‐L‐Cysteine (NAC) (Abcam #Ab143032), ROS inhibitor, was dissolved in embryo water and added to CZB at final concentration of 5 mM. In vitro maturation of drug‐treated oocytes was performed in organ culture dishes.

### 5.13 Microscopy

Images were acquired with either a Zeiss 800, 510 Meta or Leica SP8 confocal microscopes equipped with a 40×, 1.30 NA oil immersion objective. For each image, optical z‐sections were obtain using 0.5µm step with zoom of 4. For comparison of pixel intensities, the laser power was kept constant for each oocyte in an experiment. All oocytes in the same experiment were processed at the same time. To determine stable kinetochore–microtubule attachment super‐resolution images were acquired using either an AiryScan module on Zeiss LSM800 or a Lightning module on Leica SP8 equipped with a 63×, 1.40 NA oil immersion objective. Images were acquired at 0.30 μm optical sections, covering the entire spindle.

### 5.14 Image analysis

All images were analyzed using ImageJ software (NIH) (Schneider et al., 2012). For analysis, z‐sections of each cell were processed by maximum z‐projection. To measure kinetochore pixel intensity of kinetochore proteins, ACA was used to define the region of interest. Threshold levels were set in WT oocytes. At least 30 individual kinetochores per oocyte were measured and the average intensity for each oocyte was calculated for these 30 measurements. Relative pixel intensity was determined by dividing the average intensity by the average intensity of all WT oocytes in the experiment. For Securin intensity, background was subtracted from cell fluorescence. The rate of Securin‐gfp destruction was calculated according to (Nabti et al., 2017).

### 5.15 Statistical analysis

One‐way ANOVA and Student's t‐test, as indicated in figure legends, were used to evaluate the differences between groups using GraphPad Prism. The differences of *p *< 0.05 were considered significant.

## CONFLICT OF INTEREST

The authors have no conflicts to disclose.

## AUTHOR CONTRIBUTIONS

KS conceived of the study, wrote and edited the manuscript. CB, ALN, MA conducted experiments, analyzed the data, and wrote and edited the manuscript.

## Supporting information

Fig S1

Fig S2

Fig S3

Supplementary Material

## Data Availability

Data sharing is not applicable to this article as no datasets were generated or analyzed during the current study.
