# Supplementary figures and images for: Age‐dependent integrity of the meiotic spindle assembly checkpoint in females requires Aurora kinase B
Source: Aging Cell. 2021 Oct 26;20(11):e13489. doi: 10.1111/acel.13489 (PMC8590096; doi:10.1111/acel.13489)

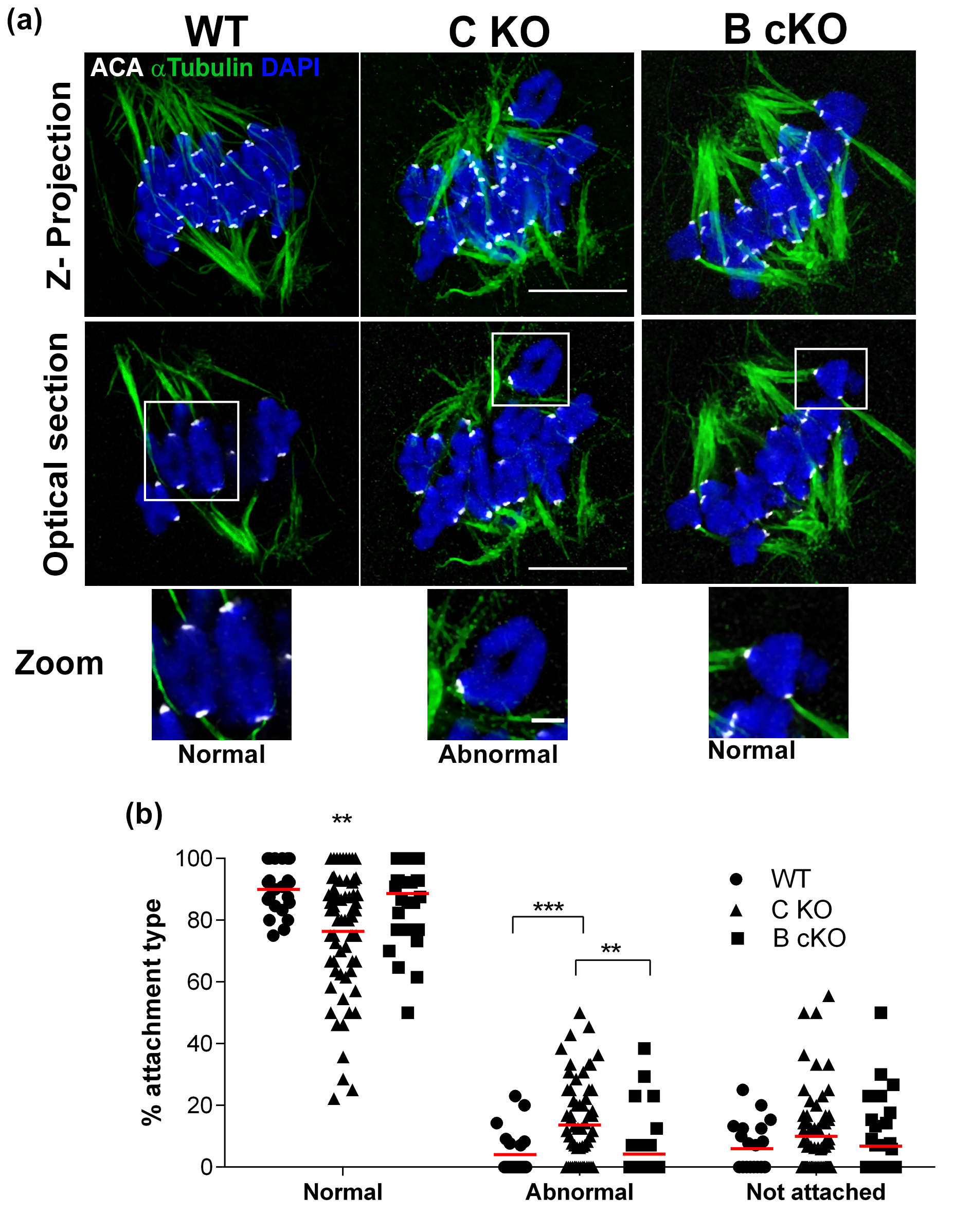

Supplement: Supplementary file 1 — Fig S1 [file ACEL-20-e13489-s003.tif]

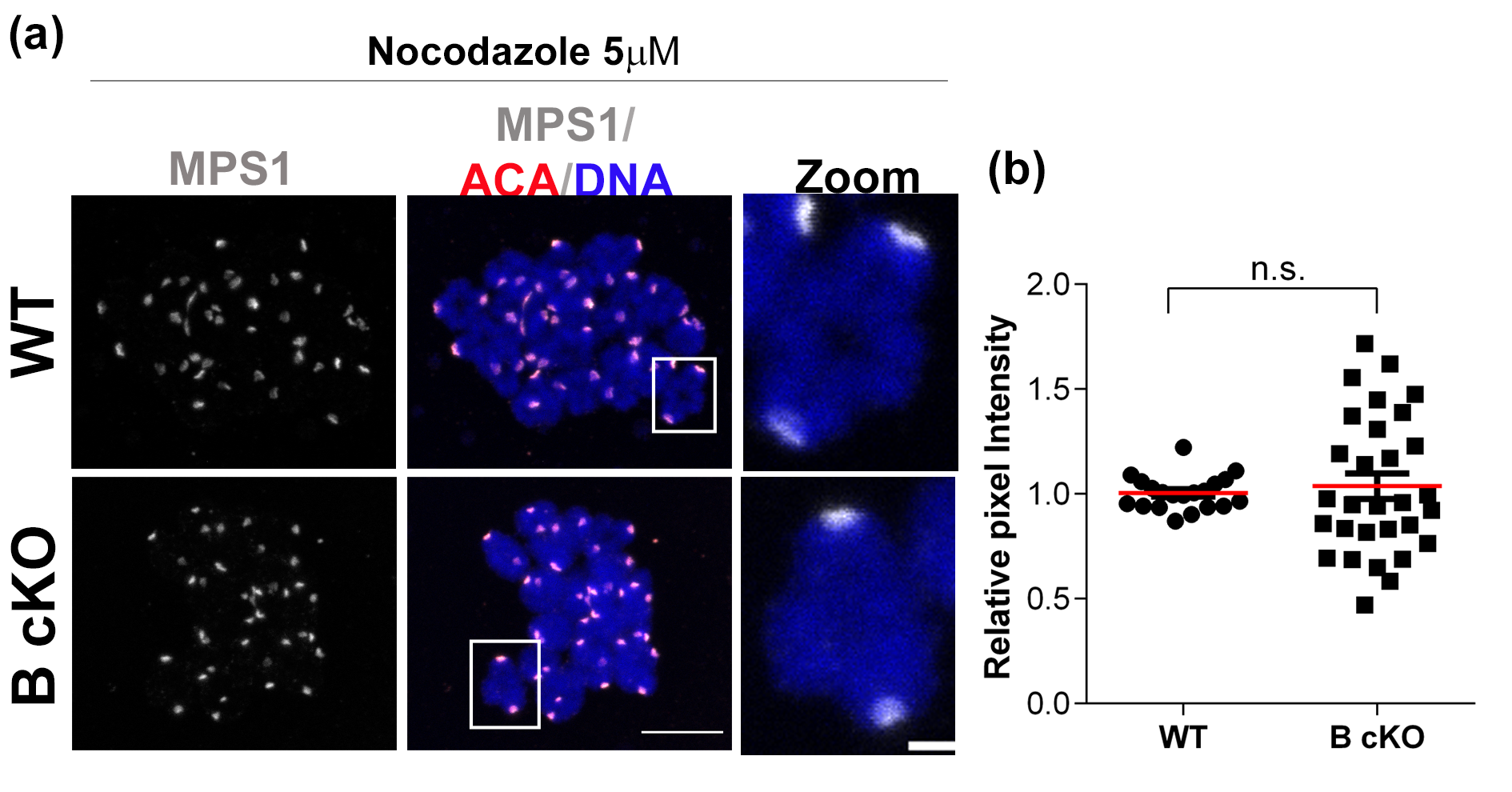

Supplement: Supplementary file 2 — Fig S2 [file ACEL-20-e13489-s001.tif]

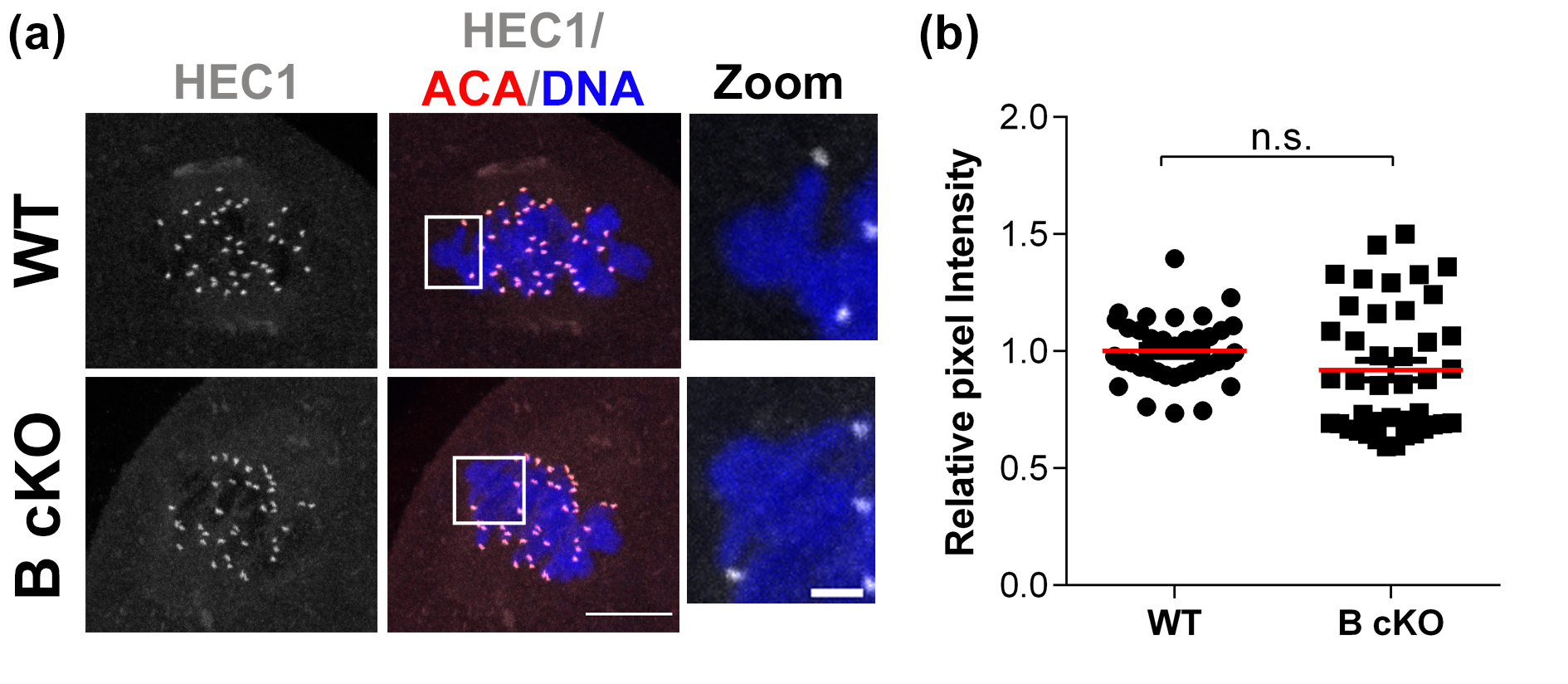

Supplement: Supplementary file 3 — Fig S3 [file ACEL-20-e13489-s002.tif]
