# Supplementary material for: Age‐dependent integrity of the meiotic spindle assembly checkpoint in females requires Aurora kinase B
Source: Aging Cell. 2021 Oct 26;20(11):e13489. doi: 10.1111/acel.13489 (PMC8590096; doi:10.1111/acel.13489)
Supplement: Supplementary file 4 — Supplementary Material [file ACEL-20-e13489-s004.docx]

**Figure S1. *Aurkb* cKO oocytes from older females have normal kinetochore- microtubule attachments at metaphase I**

(a) Representative confocal images of K-MT attachments of cold-treated spindles from oocytes at metaphase I from wild-type (WT), Aurkc knockout (C KO) or conditional *Aurkb* knockouts (B cKO) immunostained with anti-centromeric antigen (ACA) (gray), α-Tubulin (green) and DAPI (blue). Attachments were classified as: 1) normal, when homologous chromosome kinetochores were bound to MTs from opposite poles; 2) abnormal, when homologous chromosome kinetochores were bound to MTs from the same pole or when sister chromatid kinetochores were bound to MTs from opposite poles or; 3) not attached, when no MT interfaced with a kinetochore. Examples of normal and abnormal attachments of individual bivalent (boxes) are magnified and shown in zooms. (b) Quantification of % of different types of attachments in (a) (number of oocytes examined, WT: 20, B cKO: 37, C KO: 66; 3 mice/genotype). Graphs show individual oocyte values plus the mean ± SEM from 3 experiments. Scale bars: 10μm and 2μm.

**Figure S2. *Aurkb* cKO oocytes from older females recruit normal levels of MPS1 to kinetochores in presence of nocodazole**(a) Representative confocal images of chromosomes spreads at late pro-metaphase I immunostained with antibodies against ACA (red) and MPS1 (gray) from wild-type (WT) or *Aurkb* cKO (B cKO) oocytes from older females cultured in nocodazole. Examples of individual bivalents (boxes) are magnified and shown in the zoom. (b) Quantification of MPS1 intensity at kinetochores of (a) (Unpaired t Test, two-tailed, p=0.6747; number of oocytes examined, WT: 20, B cKO: 30; 3 mice/genotype). Graphs show individual oocyte values plus the mean ± SEM from 3 experiments. Scale bars: 10μm and 2μm.

**Figure S3. *Aurkb* cKO oocytes from older females have normal levels of HEC1 at kinetochores**

(a) Representative confocal images of metaphase I oocytes from wild-type (WT) or *Aurkb* cKO (B cKO) oocytes from older females immunostained with antibodies against ACA (red) HEC1 (gray). Examples of individual bivalent (boxes) are magnified and shown in the zoom. (b) Quantification of HEC1 intensity at kinetochores showed in (a) (Unpaired Students t-Test, two-tailed, p=0.1026; number of oocytes, WT: 37, B cKO: 42; 3 mice/genotype). n.s.: not significant. Graph shows the mean value per oocyte of at least 30 kinetochores measured for each oocyte plus the mean ± SEM from 3 experiments. Scale bars: 10μm and 2μm.
